# Evolutionary history reveals information on the functionality of ear tufts in owls (family: Strigidae)

**DOI:** 10.1186/s12983-025-00587-x

**Published:** 2025-11-13

**Authors:** Adrian Surmacki, Piotr Minias

**Affiliations:** 1 Department of Avian Biology and Ecology, Faculty of Biology, Adam Mickiewicz University, Uniwersytetu Poznańskiego 6, 61-614 Poznań, Poland; 2 Department of Biodiversity Studies and Bioeducation, Faculty of Biology and Environmental Protection, University of Łódź, Banacha 1/3, 90-237 Lódź, Poland

**Keywords:** Circadian rhythm, Coevolution, Crypsis, Feathers, Mimicry, Mobbing, Morphology, Predation, Phylogenetic tree, Startle displays

## Abstract

**Background:**

Ear tufts are plumage features which have particularly high prevalence in owls (Strigidae). Several hypotheses have been developed to explain their function, mostly including camouflage, species recognition, deterring predators/mobbers, and visual signaling among conspecifics. In the present study, we used phylogenetically-informed comparative approach to reconstruct evolutionary history of ear tufts across the entire Strigidae family (184 species). Specifically, data on the occurrence and relative size of ear tufts compiled from color plates and photographs were analyzed in relation to life history and ecological traits.

**Results:**

We found that ear tuft occurrence coevolved with circadian activity rhythm and predominated in species with strictly nocturnal activity. The highest evolutionary rate was found for transitions from nocturnal to mixed activity in species without ear tufts and from mixed towards nocturnal activity in species with ear tufts. Consistently, strictly nocturnal owl species showed larger ear tufts (controlling for differences in body size) compared to species with mixed activity. We also found that owls preying upon birds had relatively larger ear tufts. Finally, a strong phylogenetic signal in tuft occurrence provided evidence for high evolutionary conservedness of this trait.

**Conclusions:**

Our results suggest that ear tufts may enhance camouflage of nocturnal owls during the daylight rest, when they might be threatened by visually oriented predators or mobbed by their potential prey. Our results lay foundations for further experimental research required to determine the ultimate function of ear tufts in owls.

**Supplementary Information:**

The online version contains supplementary material available at 10.1186/s12983-025-00587-x.

## Background

Ear tufts, also called false ears or horns, are bunches of feathers located symmetrically on head in many bird orders, including Passeriformes (e.g. Horned Lark *Eremophila alpestris*), Galliformes (e.g., Common Pheasant *Phasianus colchicus*), Suliformes (e.g., Double-crested Cormorant *Nannopterum auritum*), Chardriiformes (e.g., Tufted Puffin *Fratercula cirrhata*), Podicipediformes (e.g., Black-necked Grebe *Podiceps nigricollis*) and Sphenisciformes (e.g., Macaroni Penguin *Eudyptes chrysolophus*) [7]. There is, however, a great inter-specific variation in the size and shape of these structures [7]. Species with particularly long tufts (similar to plumes) usually keep them draped along the head. In some cases, birds erect ear tufts, especially during courtships or antagonistic interactions (e.g., [4, 26]).

Ear tufts in owls Strigidae are particularly conspicuous and several hypotheses have been proposed to explain their function. First, ear tufts may enhance camouflage (*camouflage hypothesi*s, [35, 45]). This was supported by the observations that some owls erect their tufts in response to approaching danger [35]. An erection of ear tufts often coincides with changes in body posture from rounded to vertically elongated [35]. This behavior, combined with a cryptic coloration, might be considered advantageous, because it provides concealment against visually oriented predators or mobbers [35]. Alternatively (but not exclusively), it has been suggested that ear tufts act as head ornamentation used in visual communication within species (*intraspecific communication hypothesis* [18]) or between species (*interspecific communication hypothesis* [18]). Intraspecific signals conveyed by ear tufts may help to localize individuals in dim light, act as a sexual signal, aid recognition of kin and its emotional state [18, 35]. In contrast, interspecific signals may be used as confusion or startle displays (i.e., by a sudden tuft erection), which may intimidate potential predators or mobbers [18]. Finally, ear tufts may deter mammalian predators by making owl head appearance similar to carnivores like lynx, fox or marten [28].

To date, only the camouflage hypothesis has been tested experimentally in owls. Three captive Northern Pygmy Owl (*Glaucidium californicum*) were confronted with the domestic cat (*Felis catus*) or the Peregrine Falcon (*Falco peregrinus* [22]). Owls consistently responded with “concealment posture”, which included erected ear tufts [22]. There were also two attempts to explain functionality of ear tufts using a comparative approach. First, an inspection of tufted and non-tufted owl species frequencies suggested that the evolution of this trait may have been associated with nocturnal activity, but not with abundance of mammalian predators [35]. While these observations may provide support the camouflage and species recognition hypotheses (but not the predator mimicry hypothesis [35]), the data were not subject to any formal statistical analysis and thus, the hypotheses still need testing using a rigorous phylogenetically-controlled approach to draw robust conclusions. More recently, Galeotti and Rubolini [18] studied associations of head ornamentation in owls with body size, activity rhythm and habitat type. In total, nine ornamental traits were scored, including ear tuft size, all showing positive inter-correlations. It was found that head ornamentation was stronger in diurnal species which live in more open habitats [18]. It was concluded that head ornamentation (including ear tufts) could have evolved in accordance with the *intraspecific* rather than *interspecific communication hypothesis* [18]. Although phylogenetic relationships between species were taken into account by comparing pairs of congeneric taxa with contrasting level of ornamentation and originating from the same geographic region, the final sample size was limited (n = 35 species pairs [18]). Also, the conclusion on the stronger head ornamentation in diurnal owl species seems to be inconsistent with early observations by Perrone [35], suggesting more conspicuous ear tuft in owls with strictly nocturnal activity. Taking all this into account, the hypotheses on ear tuft functionality still require a thorough investigation. Moreover, neither of these two studies attempted to reconstruct evolutionary history of ear tuft occurrence in owls and, thus, we lack information on the ancestral state, transition rates and evolutionary lability of this trait.

Despite limited empirical evidence, hypotheses related to the camouflage seem to provide the most plausible explanations for the functionality of owl ear tufts. Virtually all owl species have a drab and mottled plumage [8], which was likely to have evolved directionally to match the coloration of environmental background (e.g., the bark of trees), as found in other avian species with similar type of plumage [46]. Ear tufts may additionally increase owl concealment by disrupting their body outline or mimicking broken branches [13]. Camouflage by ear tufts should be favored by selection in typically nocturnal species, which sleep during the day, and thus are less vigilant to avoid diurnal predators, including mammals and birds of prey [13]. Resting owls are also threatened by mobbers—usually small passerines that owls may prey on [22, 29, 32]. Even if mobbing usually does not pose a physical threat to owls (but see [29]), it should be regarded as costly and disadvantageous, because it may lead to changes in habitat use and diet [16, 29, 32, 34]. Moreover, mobbing can have significant physiological consequences, such as an increased stress level or disruption of the circadian rhythm due to increased vigilance [16]. While both diurnal and nocturnal owls may suffer from mobbing, many small diurnal owl species specialize in hunting passerines [13, 29]. Thus, diurnal owls may use camouflage strategies not only to protect themselves from predators and mobbers, but also to enhance hunting success.

Here, we aimed to reconstruct evolutionary history of ear tuft occurrence in owls and to test for co-evolutionary associations of this trait using a rigorous phylogenetically-informed approach and a robust phylogenetic coverage (the entire Strigidae family). We primarily expected that if ear tufts evolved to aid concealment against visually oriented predators and mobbers, they should occur more frequently (and be more expressed) in nocturnal species that rest during the day. Additionally, the expression (size) of ear tufts may be enhanced in species which have birds in their diet.

## Methods

### Ear tufts

We collected data on ear tufts occurrence and size for Strigidae owls from color plates available in the Handbook of Birds of the World (hereafter HBW; [8]) and photographs mainly deposited at Macaulay Library (Cornell Lab of Ornithology, Cornell University, https://www.macaulaylibrary.org; detailed information on the origin of all photographs is presented in Additional file 1). Illustrations of birds published in HBW have been based on a wide selection of photographs of live birds, museum skins and captive birds, all presented in a scale [8]. Thus, this source is traditionally appreciated for high quality and reliability of illustrations and has been used to extract information for comparative analyses of morphological traits in a wide spectrum of avian lineages, including owls (e.g., [9, 11, 33]). To analyze ear tuft occurrence, we originally compiled data for all extant members of Strigidae family illustrated in del Hoyo et al. [8] (n = 188). Four of these taxa (*Otus vermiculatus, Glaucidium cobanese, G. hoskinsii, G. tucumanum*) were classified as subspecies in more recent systematics [25]. All four taxa were removed from the final dataset and, thus, 184 owl species were retained for downstream analysis. Ear tuft occurrence was coded as a binary character (tufts present or absent), based on the representation of adult plumage. Owls are monomorphic with respect to ear tufts occurrence [8], therefore our data were representative for both sexes.

The area of ear tufts was measured using color illustrations from the HBW [8] and photographs. For both illustrations and photographs, we chose images showing owls in a similar upright pose, with both ear tufts visible. Color plates were digitalized to 300-dpi JPEG files using Epson Perfection V600 Photo scanner. We measured the area of ear tufts and the remaining body silhouette using the polygon selection tool available in the ImageJ software [44]. Since legs were often partially covered by branches, the area of body silhouettes was measured without legs across all the species. The proximal border of the tuft was determined by marking two points where the ear tuft and the body contours diverge, one on the left and the other on the right side of the tuft. The straight line connecting both points was used as the proximal border of the tuft. We recorded data on body and ear tuft area in square centimeters for color plates and in pixels for photographs. If there were multiple subspecies or color morphs per species illustrated in the HBW, the measurements were averaged to yield a single species-specific value for downstream analyses. For photograph analysis, we have selected up to five best-quality photographs per species (at the appropriate resolution and showing the owl in the most standard position, see above). On average, we measured 4.1 ± 0.2 (SD) available photographs per species and measurements from photographs depicting the same species were averaged prior to analysis. Two species (*Bubo bengalensis* and *Jubula letti*), were excluded from analysis of ear tuft size, because they were depicted with only one tuft or tufts folded, respectively. The final dataset comprised information on ear tuft area for 92 (HBW color plates) and 81 (photographs) species.

To assess reliability of our tuft size measurements, we correlated measurements from HBW color plates and photographs. Since the two measurements were on different scales (cm^2^ and px, respectively), we used the ratio of tuft area to the total body surface area. We found that both measurements were significantly correlated (r = 0.53, *p* < 0.001, n = 81), suggesting that HBW color plates provided reliable information on the relative size of the ear tufts.

### Morphological, ecological and biogeographical predictors

Based on information provided by del Hoyo et al. [8] and Birds of the World (https://birdsoftheworld.org), we compiled data on the following morphological and ecological traits: body size, circadian activity, habitat, and diet. The body size was quantified as the total body length reported for the species. Minimal and maximal values of each reported range were averaged. The circadian activity rhythm was classified as either nocturnal, i.e. active mainly during the night, or mixed, i.e. active mainly during daytime or at both night and either day, dusk or dawn. All species were assigned to one of two broad-scale habitat categories: forest or non-forest. The first one referred to typical woodlands with a dense canopy cover, while non-forest category comprised a wide spectrum of habitats, including rocky areas, deserts, tundra, grasslands, savannahs, and human settlements. The diet also consisted of two categories, with birds either present or absent in the diet as a food source. Finally, we compiled biogeographical data on species occurrence. Breeding latitude was measured as a geometric centroid of breeding range from BirdLife International distribution maps [1]. Geometric centroids for breeding and resident spatial polygons were computed using *gCentroid* function in the *rgeos* package [2] developed for R statistical environment (R Foundation for Statistical Computing, Vienna, Austria), following methodology proposed by Vincze [48].

### Phylogenetic tree

Phylogenetic relationships between species were primarily reconstructed using the complete time-calibrated avian phylogeny available at the BirdTree webserver [25]. We retrieved 1000 alternative trees (following methodology by Rubolini et al. [40]) with a backbone topology from Ericson et al. [15] and summarized them into a single consensus tree (henceforth referred to as BirdTree phylogeny) using Geneious v. 10.0.5 (Biomatters Ltd, Auckland, New Zealand) software. This phylogeny included all our study species of owls (n = 184) and its topology well corresponded with recent Strigidae phylogeny developed by Salter et al. [42], revealing the ancestral clade containing several major genera (e.g. *Aegolius*, *Athene*, *Glaucidium*, and *Ninox*) and the more derived clade containing all the remaining major genera (e.g. *Asio*, *Bubo*, *Megascops*, *Otus*, and *Strix*). At the same time, we acknowledged that BirdTree topology was originally generated combining species with and without genetic information [25], which could have introduced phylogenetic uncertainty in our models. Thus, to increase reliability of our inferences, we re-ran phylogenetically-informed MCMCglmm models (see below for details) using more recent avian supertree developed by McTavish et al. [27]. This tree provided information on phylogenetic relatedness of 179 owl species from our dataset and is henceforth referred to as the McTavish et al. phylogeny.

### Phylogenetic signal

Phylogenetic signal was calculated as Fritz and Purvis’ D, which was designed for binary traits [17]. Briefly, D is scaled using two null distributions of (1) random expectation, where character states are randomly distributed among species without respect to phylogeny, and (2) expectation under the Brownian Motion evolution, which is consistent with purely neutral evolution (trait changes only due to genetic drift) or fluctuating selection [20]. Expected distributions of D under these two evolutionary scenarios were simulated with 1000 permutations using BirdTree phylogeny. Significance of the observed D was tested against the means expected under the random (D = 1) and Brownian Motion (D = 0) distribution. Low D values indicate that a trait is phylogenetically conserved (D < 0 indicates higher phylogenetic conservancy than expected under the Brownian Motion evolution), while high D values indicate that a trait is phylogenetically labile (D > 1 indicates that a trait is phylogenetically overdispersed when compared to the random expectation). Computations of phylogenetic signal were conducted in the *caper* R package [30].

### Evolutionary reconstruction

To reconstruct evolutionary history of ear tuft occurrence in owls we first fitted two alternative models assuming asymmetrical (all-rates-different, ARD) and symmetrical (equal-rates, ER) transition rates between the binary character states (i.e. tufts present and absent). Both models were fitted using *fitMk* function from the *phytools* R package [39] and compared with Akaike Information Criterion (AIC). The best-fitting model was used to quantify transition rates and a stationary distribution of state frequencies was used to estimate the ancestral state at the root node. We also used stochastic character mapping [24] to infer posterior probabilities of character states at each node of the phylogeny and the total number of forward and backward transitions between character states (tuft gain and loss, respectively). For this purpose, we used MCMC approach to simulate, sample and summarize 1000 unambiguous histories of ear tuft evolution from their posterior probability distribution using the *make.simmap* function from the *phytools* R package. As we primarily hypothesized that ear tuft occurrence should be associated with activity rhythm, we also conducted stochastic character mapping for the nocturnal vs. mixed activity in owls. Posterior probability distributions of both ear tuft occurrence and activity rhythm were plotted on the BirdTree phylogeny using *phytools* R package and visually compared.

### Phylogenetically-informed analyses

In the first step, we used BayesTraits v.3.0 software [31] to test our primary hypothesis on the association of ear tuft occurrence with circadian rhythm. Coevolution of both traits was tested by fitting discrete independent and dependent models of evolution using maximum likelihood approach. The dependent evolution model was constrained, so that both transition rates (backward and forward) between selected pairs of binary states did not vary. We ran all possible combinations of constrained dependent models and identified the best-fitting model using log-likelihood (Lh) comparisons and the likelihood ratio test. Next, we used the best fitting (dependent) model to estimate transition rates between pairs of binary states of ear tuft occurrence and activity rhythm. All models were run using all 1000 alternative phylogenies retrieved from BirdTree webserver to account for phylogenetic uncertainty.

In order to identify predictors of ear tuft expression (size) we also fitted Bayesian Phylogenetic Mixed Models using the *MCMCglmm* R package [19]. These models were limited to species in which tufts were recorded. Alternative models were fitted using: *i*) measurements originating from two different sources, i.e., color plates (n = 92 species) and photographs (n = 81 species), and ii) two different phylogenies (BirdTree and McTavish et al. [27]). This framework resulted in four different models fitted. In all the models, we included circadian activity rhythm (night vs. mixed), habitat (forest vs. non-forest) and diet (birds present vs. absent in diet) as binary fixed factors, and breeding latitude was entered as a covariate. We also included log body size (total body area) as another covariate in all the models. All the models were fitted using Gaussian distribution of the response variable and uninformative priors (variance set to 1, belief parameter set to 0.002). Each model was fitted using 200,000 iterations, thinning value of 100, and a burn-in period of 100,000, yielding an expected sample size of 1000 iterations, which were used as a posterior distribution for parameter estimates. The proportion of variance explained either by the random effect (phylogeny) or random/fixed effects was calculated as marginal and conditional R^2^, respectively, using *pseudoR2.MCMCglmmm* function from the *evoldiver* R package [41].

## Results

**Fig. 1:**
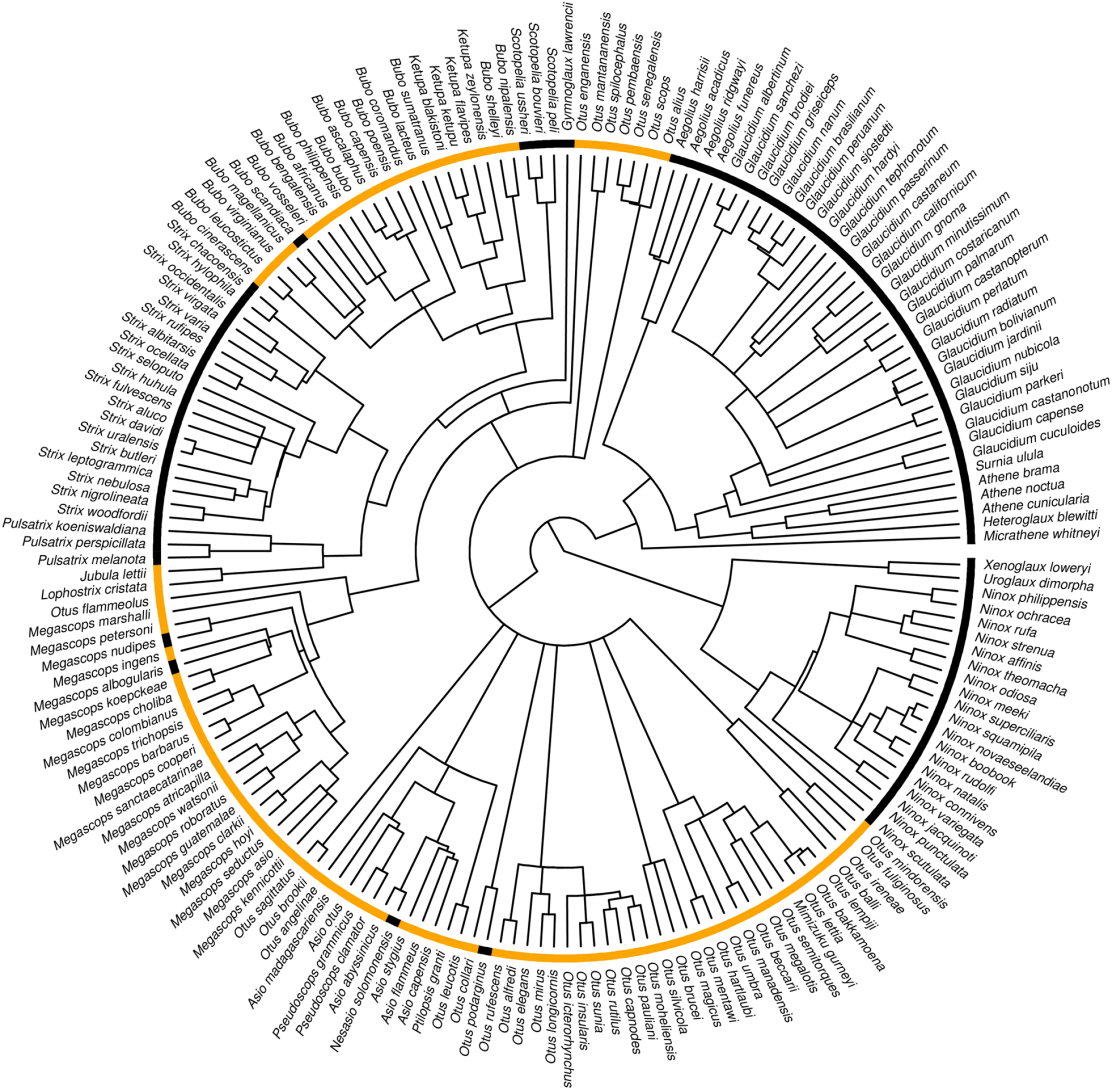
Phylogenetic distribution of ear tuft occurrence across the phylogeny of owls Strigidae (184 species). Ear tuft presence (yellow) or absence (black) is indicated at each terminal node

**Fig. 2:**
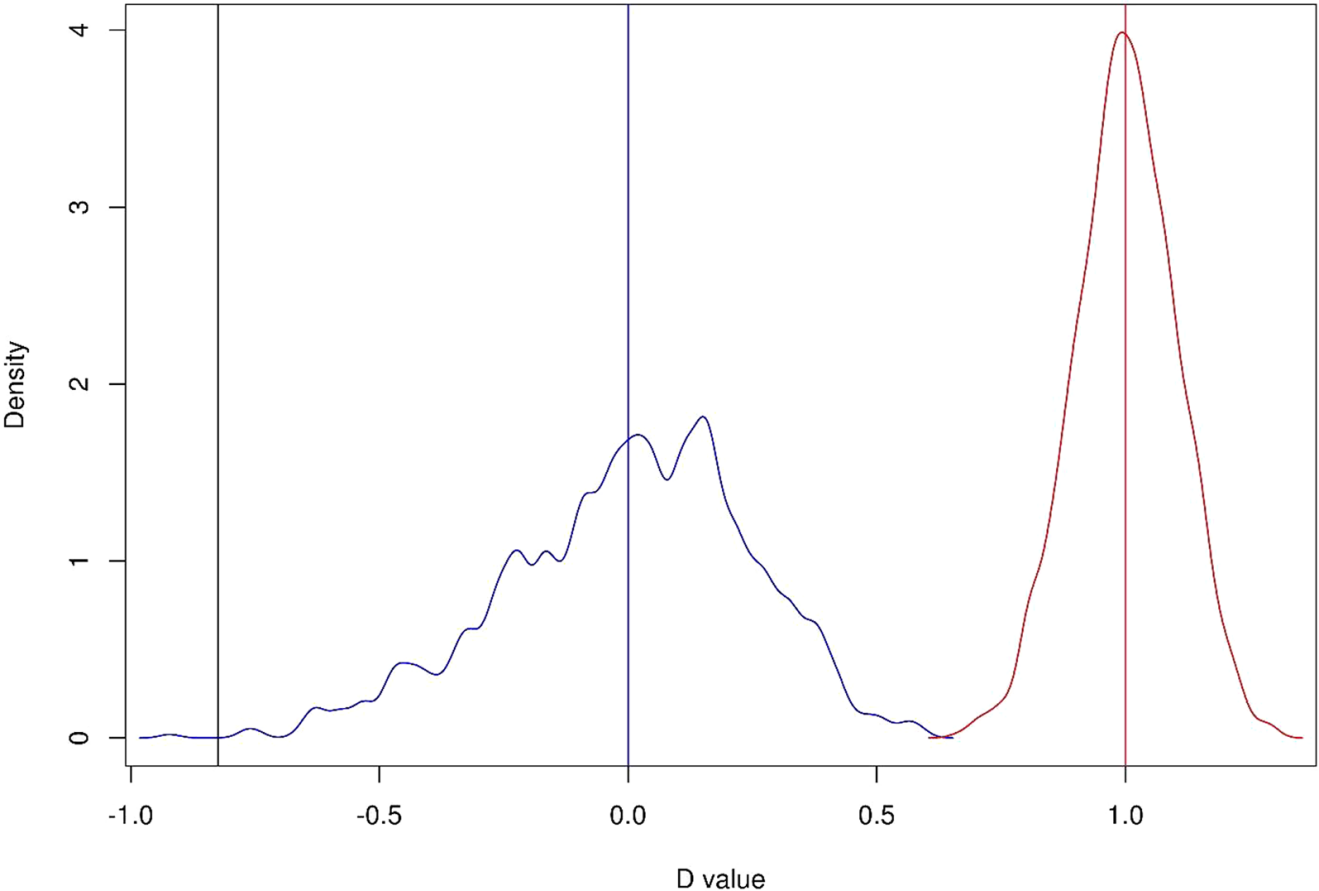
Phylogenetic signal, as measured with Fritz and Purvis’ D, in ear tuft occurrence in owls Strigidae (184 species). Observed D value is marked with grey line, while means expected under random (D = 1) and Brownian Motion (D = 0) evolution are marked with thin red and blue lines, respectively. Distribution densities of D values expected under random and Brownian Motion evolution (as simulated with 1000 permutations) are shown with red and blue curves, respectively

**Fig. 3:**
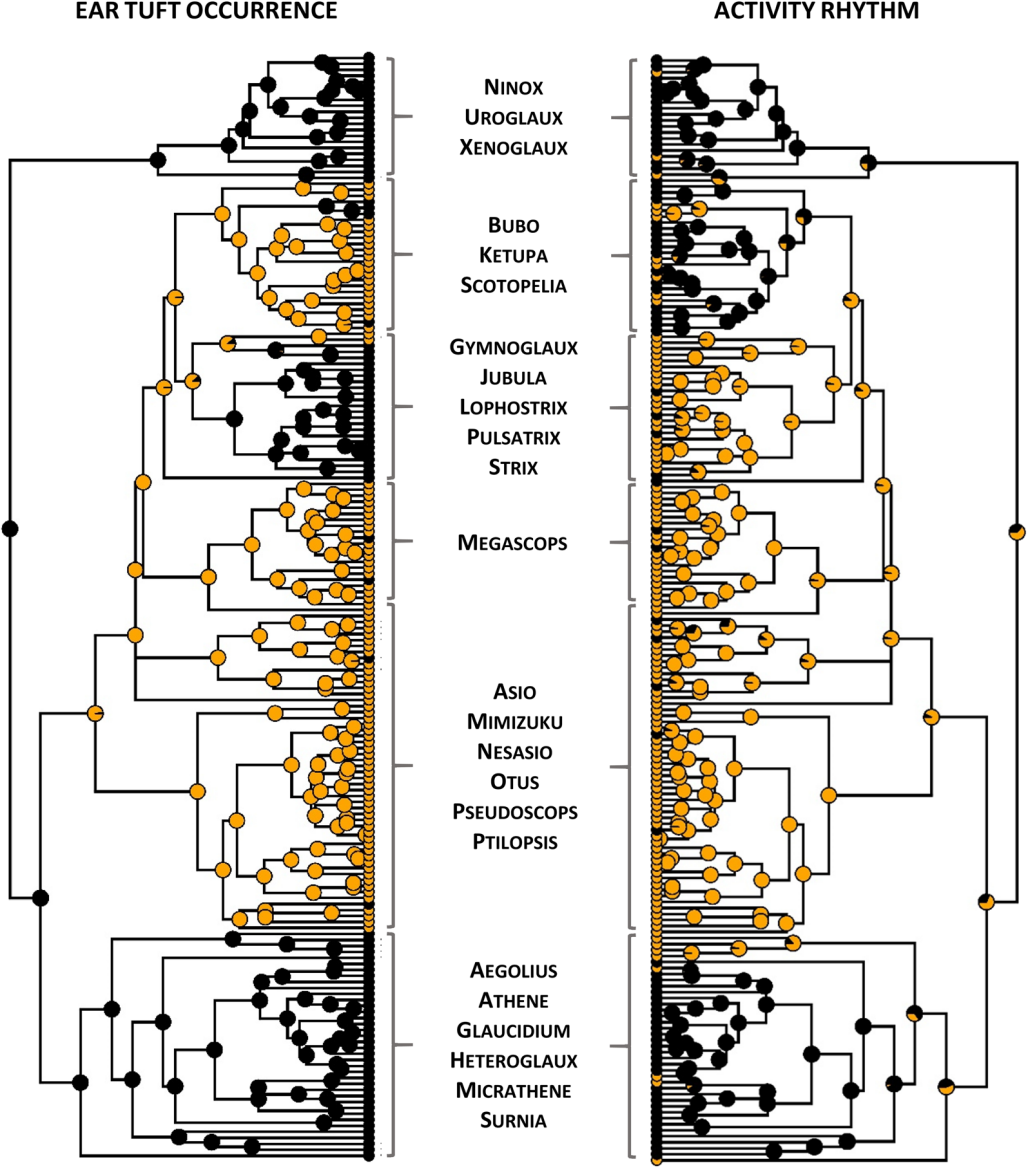
Evolutionary reconstruction of ear-tuft occurrence (left panel) and activity rhythm (right panel) across the phylogeny of owls Strigidae (184 species). Character states are shown at terminal nodes, while posterior probabilities of character states (estimated with stochastic character mapping) are shown at each internal node. Ear tufts were marked as either present (yellow) or absent (black), while activity rhythm was marked as either strictly nocturnal (yellow) or mixed (black)

Ear tufts were recorded in 51.1% (94 of 184) of owl species and in 37.0% (10 of 27) of genera (Fig. 1). There was a strong phylogenetic signal in ear tuft occurrence across owls (*D* = − 0.83), being significantly different from the random expectation (*D* = 1, *P* < 0.001). The negative *D* values indicated that ear tuft occurrence was more phylogenetically conserved than the expectation under the Brownian Motion evolution (*D* = 0, Fig. 2). The reconstruction of ear tuft evolution provided support for asymmetric backward and forward transition rates between binary character states (i.e., tuft presence and absence), as indicated by the better fit of all-rates-different (ARD) over equal-rates (ER) model (AIC: 80.63 vs. 86.26). Under the ARD model, ear tuft absence was unequivocally identified as the ancestral state at the root of Strigidae phylogeny (probability: 1.00; Fig. 3) and most simulations indicated for a single evolutionary event of tuft gain relatively early in owl evolution (on average 1.28 gain events across 1000 simulations; Fig. 3). After their first evolutionary appearance, tufts were retained in most descendant lineages, but we also observed tuft loss in certain taxa (on average 9.27 loss events across 1000 simulations; Fig. 3) and, thus, backward transition rates (tuft loss) dominated over forward transition rates (tuft gain) during the evolution of the entire clade (0.0110 vs. 0.0014 for backward and forward transitions, respectively). Visual comparison of posterior probabilities of ear tuft occurrence and activity rhythm showed strong consistency in the phylogenetic distribution of ear tufts and nocturnal activity across owl phylogeny (Fig. 3).

**Fig. 4:**
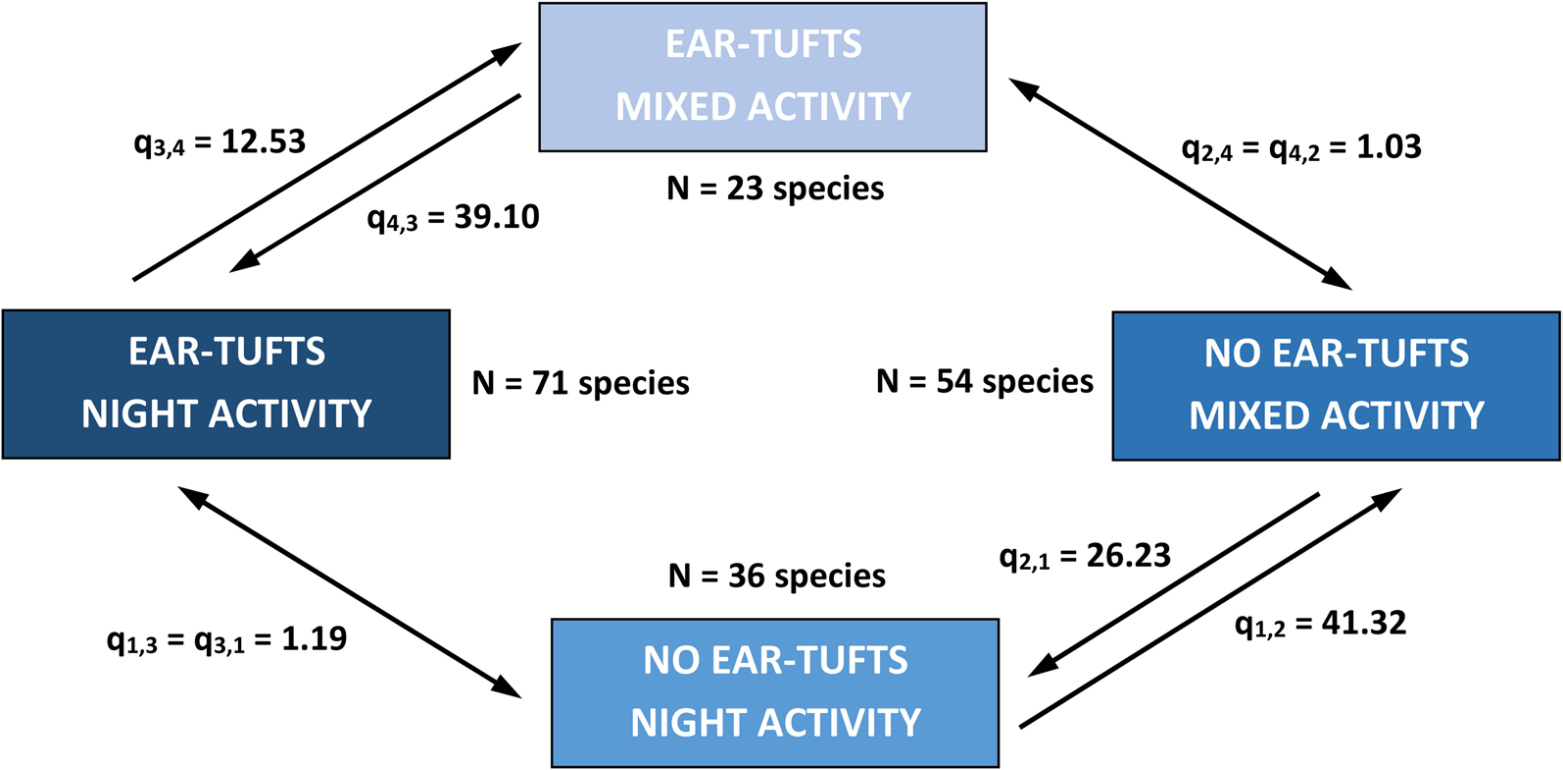
Evolutionary transition rates (q) between pairs of binary states of ear tuft occurrence and circadian activity rhythm, as assessed with restricted best-fitting dependent evolution model. Gradient of blue coloration reflects frequency (no. species) of state pairs of tuft occurrence and activity

Coevolution of ear tuft occurrence and activity rhythm was supported by significantly higher log-likelihood (Lh) of the best-fitting dependent vs. independent model of evolution of these traits (Lh = − 152.2 vs. Lh = − 165.4; LR = 26.4, *P* < 0.001). The best-fitting dependent evolution model revealed the highest rates for the transitions from nocturnal to mixed activity in species without ear tufts (q_1,2_ = 41.32) and from mixed towards nocturnal activity in species with ear tufts (q_4,3_ = 39.10). The rate of the backward transitions was markedly lower (towards nocturnal activity in species without ear tufts: q_2,1_ = 26.23; towards mixed activity in species with ear tufts: q_3,4_ = 12.53) (Fig. 4). Forward and backward transition rates between ear tuft character states (presence and absence) were low and symmetrical (q_1,3_ = q_3,1_ = 1.19 and q_2,4_ = q_4,2_ = 1.03; Fig. 4), being consistent with strong evolutionary conservedness of this trait.

**Fig. 5:**
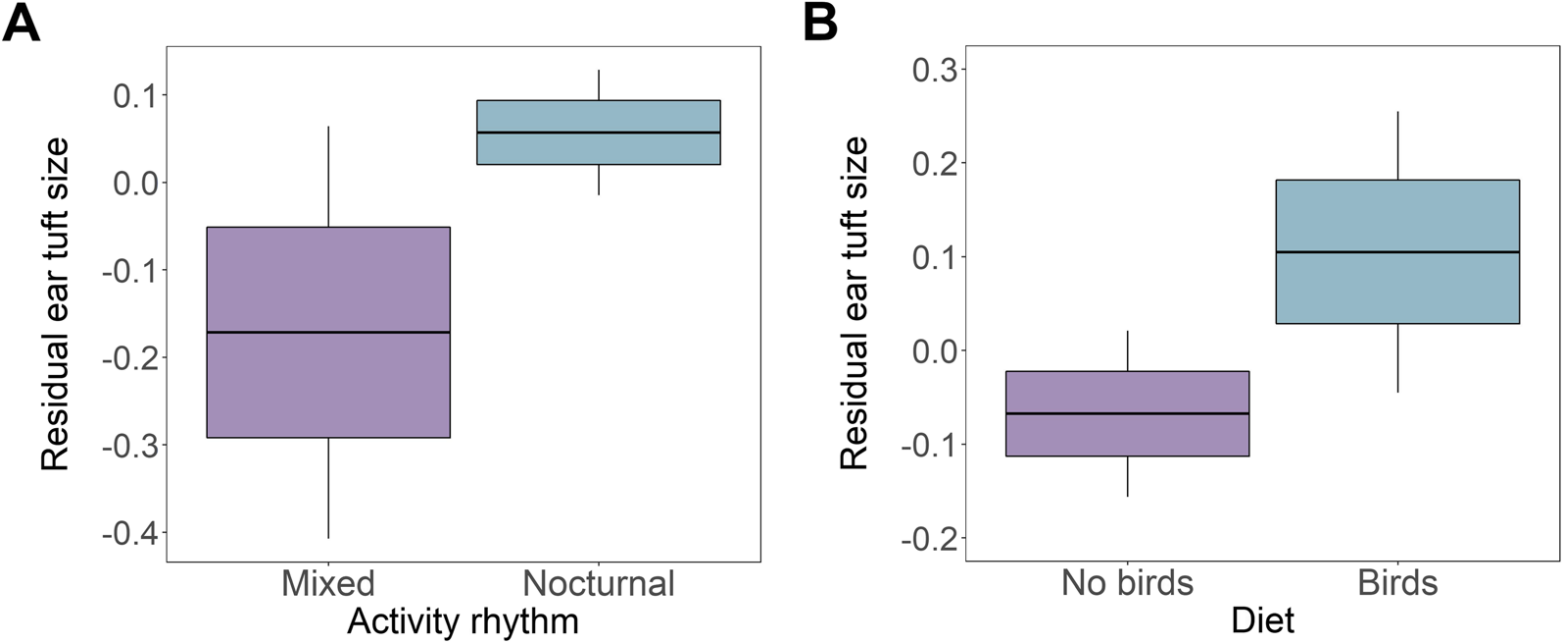
Associations of residual ear tuft size (area) with activity rhythm (**A**) and diet (**B**) in owls Strigidae. Central line—mean, box—SE, whiskers—95% confidence interval

Consistently, our phylogenetically-informed models restricted to owl species with ear tufts identified activity rhythm as a major predictor of tuft area and, specifically, we found that nocturnally active species had larger tufts (Table 1, Fig. 5). Significance of this association was retained across different sources of measurements (i.e., color plates and photographs) and phylogenies (i.e., BirdTree and McTavish et al. trees) (Table 1). Apart from activity rhythm, we identified diet as a significant predictor of ear tuft size, as species with birds in diet had significantly larger ear tufts compared to species with no birds in diet (Table 1, Fig. 5). This association was apparent in the analyses based on tuft size measurements originating from both sources (color plates and photographs) while using BirdTree phylogeny. However, in the analyses based on McTavish et al. [27] phylogeny this effect retained significance only for photograph measurements (Table 1). No evidence was found for the effects of habitat and latitude on ear tuft size in owls (Table 1).

**Table 1:** The results of Bayesian phylogenetic mixed models for ear tuft size (area) in owls Strigidae

Source	Predictors	BirdTree phylogeny				McTavish et al. phylogeny			
		Estimate	Lower 95% CI	Upper 95% CI	*P*	Estimate	Lower 95% CI	Upper 95% CI	*P*
Color plates	Intercept	**− 3.22**	**− 3.91**	**− 2.53**	** < 0.001**	**− 3.87**	**− 5.10**	**− 2.46**	** < 0.001**
	Activity	**− 0.38**	**− 0.61**	**− 0.13**	**0.002**	**− 0.37**	**− 0.60**	**− 0.14**	**0.002**
	Habitat	0.07	− 0.08	0.27	0.440	− 0.03	− 0.18	0.16	0.732
	Diet	**0.29**	**0.06**	**0.51**	**0.014**	0.13	− 0.08	0.37	0.250
	Latitude	− 0.001	− 0.008	0.049	0.744	0.001	− 0.005	0.007	0.760
	Log body area	**0.90**	**0.78**	**1.05**	** < 0.001**	**1.02**	**0.81**	**1.23**	** < 0.001**
		Marginal R^2^: 0.79, conditional R^2^: 0.83				Marginal R^2^: 0.68, conditional R^2^: 0.94			
Photographs	Intercept	**− 4.25**	**− 6.26**	**− 2.41**	** < 0.001**	**− 4.34**	**− 6.36**	**− 1.95**	** < 0.001**
	Activity	**− 0.43**	**− 0.77**	**− 0.11**	**0.012**	**− 0.45**	**− 0.75**	**− 0.06**	**0.008**
	Habitat	0.02	− 0.24	0.28	0.858	0.03	− 0.22	0.29	0.828
	Diet	**0.51**	**0.23**	**0.82**	** < 0.001**	**0.47**	**0.14**	**0.78**	**0.008**
	Latitude	− 0.004	− 0.013	0.005	0.434	− 0.003	− 0.013	0.007	0.488
	Log body area	**1.04**	**0.90**	**1.18**	** < 0.001**	**1.05**	**0.89**	**1.19**	** < 0.001**
		Marginal R^2^: 0.71, conditional R^2^: 0.77				Marginal R^2^: 0.69, conditional R^2^: 0.76			

The models were run using measurements from either color plates or photographs and phylogenetic relationships were reconstructed based on either BirdTree or McTavish et al. [27] phylogeny. Significant (*P* < 0.05) predictors are marked in bold. Marginal and conditional R^2^ values are provided for each model

## Discussion

Our reconstructions of evolutionary history in owls Strigidae identified ear tuft occurrence as a highly conserved trait, but also supported tight coevolution of ear tufts with circadian activity. In fact, the highest evolutionary rate was found for transitions from nocturnal to mixed activity in species without ear tufts and from mixed towards nocturnal activity in species with ear tufts. Nocturnal owl species also had larger ear tufts compared to species with mixed activity. Our results seem to provide a robust formal support for the camouflage hypothesis formulated based on the early observations by Perrone [35], but at the same time they showed inconsistencies with evolutionary patterns described for the multi-trait head ornamentation in owls [18].

The most likely explanation for the coevolution of ear tufts with activity rhythm is that head appendices increase the efficiency of owl concealment during day time rest. We may assume that species which sleep during the day are less vigilant and, thus, more prone to detection by diurnal visually oriented predators. There are two possible non-exclusive mechanisms in which ear tufts may provide camouflage for resting owls. The first one is associated with a mimicry (or a masquerade) to a dead snag on a tree. This strategy is commonly adopted by potoos Nyctibiidae and frogmouths Podagridae, which, like many owls, are strictly nocturnal and rest during the day [21]. Second, ear tufts, combined with plumage pattern, may enhance owl concealment by coincident disruptive coloration. This concealment mechanism creates false “internal borders” on animal body, which may reduce visibility of its most conspicuous parts, e.g., eyes or legs [6]. In many owl species, ear tufts and eyebrows are similarly colored and form a V or X shape contrasting sharply with the rest of facial disc (e.g., Crested Owl *Lophostrix cristata* and Barred Eagle-owl *Bubo sumatranus*, [8]). Such a pattern may either help to directly hide eyes, disrupt general “face” appearance, or both. The coincident disruptive coloration provides efficient concealment strategy, because it also breaks the outline of body silhouette, which may disrupt the so-called predator search image [12, 36, 49].

Surprisingly, the most convincing evidence for anti-predatory function of owl ear tufts comes from studies on species lacking conspicuous tufts. The Northern Pygmy Owls which were exposed to a mammalian or avian predator responded with a concealment posture, including: 1) erected tuft-like feather structures above eyebrows, 2) closed eyes, 3) feathers compressed to the body, and 4) one wing folded across the front of the body and raised to bill level [22]. Whereas pygmy owls do not have any distinct permanent ear tufts, they are capable to temporarily form a similar structure from raised eyebrow feathers [22]. A similar behavior has been observed in the European Pygmy Owl (*Glaucidium passerinum*), Elf Owl (*Micrathene whitneyi*) and Ferruginous Pygmy Owl (*Glaucidium brasilianum*, [22, 43]). Such responses have not yet been recorded in nocturnal tufted owls, but it is conceivable that they may adopt a similar camouflaging posture even during daytime rest. Previous studies suggest that owls, like many other birds, are partially vigilant when they sleep, which is primarily due to unihemispheric slow-wave sleep (USWS, [38]). The USWS occurs when one half of the brain sleeps, while the other stays alert, meaning that birds often sleep with one eye open and are fully responsive [37, 38]. Thus, it seems possible that owls sleeping with ear tufts folded may rise them (and change body posture) after detecting the potential threat (e.g., by using acoustic cues). Nocturnal owls with no ear tufts (neither permanent nor temporal ones, e.g., Saw-whet *Aegolius acadicus* and Boreal *Aegolius funereus* Owls), respond to disturbance by rising the outer crown feathers of their facial disks [5], which provide further support for the role of head appendices in anti-predatory camouflage.

While circadian rhythm was identified as the key trait driving the evolution of ear tufts in owls, we also found support for the associations of diet with ear tuft size. Specifically, owls which have birds in their diet showed more conspicuous (bigger relative to total body size) tufts. This result agrees with our prediction that ear tufts help to deter or conceal from the small passerines they prey on, which may show active mobbing behavior towards owl predators during the daytime. We suggest that both diurnal and nocturnal owl species may have evolved morphological adaptations against mobbing by their prey. Although these analyses were restricted exclusively to tufted owls, there were 28 typically nocturnal tufted species which sometimes prey on diurnal birds, e.g. there were 86 passerine species recorded in the diet of the Great Horned Owl *Bubo virgnianus* [14]. On the other hand, 19 diurnal owls which prey upon birds have ear tufts. Thus, our results suggest that this mechanism could act as additional selective force driving higher tuft conspicuousness. The association of diet with ear tuft size was apparent in most, but not all of our phylogenetically-informed models, indicating possible issues with phylogenetic uncertainty. Hence, we suggest that this result should be treated with caution.

Beside camouflage, ear tufts may also be used as a startle display or deimatic behavior, which relies on a sudden display of visually conspicuous features in the face of approaching danger [47]. It was hypothesized that the primary aim of a startle display is to scare or confuse the predator, thus giving the prey time to escape or even to thwart predator attack [47, 23]). Indeed, a concealing posture with raised tuft-like eyebrows observed in *Glaucidium* spp. upon predator approach could also act as a startle display [22, 43]. In owls, the effect of raised ear tufts may be amplified by the shift in the body posture and demonstration of bright eye iris [3, 22]. Erected ear tufts could be also used as a startle display against mobbing birds, which was supported by observations of eyebrows erection in Norther Pygmy Owls, when approached by flocks of passerines [22]. It is possible that ear tufts may have different functions depending on whether the owl has been already detected by predators/mobbers or not, either increasing crypsis or startling intruders. Whether erected ear tufts deter or provide concealment against mobbing birds requires further experimental investigation.

Our analyses of transition rates provided additional insights into the evolution of ear tuft occurrence in owls. Evolutionary reconstructions revealed that ear tuft occurrence was highly conserved and for most available phylogenies we identified only a single transition event towards tuft presence, which occurred relatively early in the evolution of Strigidae. This forward transition was clearly associated with nocturnal activity. Evolutionary maintenance of ear tuft was less conserved, as we detected nearly ten transitions towards tuft loss in descendant lineages or taxa. Finally, we identified tuft absence as the ancestral state in Strigidae, while tuft presence should be considered a derived state within this clade, reflecting specific adaptation of owls to nocturnal activity.

While we believe that our robust comparative study advances the current knowledge on the evolution of a conspicuous morphological trait in owls, it should be emphasized that our study is purely correlational, so our final conclusions should be supported by further behavioral experiments conducted in natural habitats. These could test how the presence of tufts in realistic models of owls affects their detection by wild birds (potential mobbers) or human observers. In addition, remote-controlled robotic owl models could be used to investigate whether raising tufts deters (or attracts) avian mobbers.

## Conclusions

The results of our comparative study revealed that evolution of ear tufts in owls was primarily associated with nocturnal activity, although more conspicuous (larger) ear tufts were also present in bird-hunting species. Our findings suggest that ear tufts could have evolved as a morphological adaptation, which decreases detectability by diurnal predators and mobbing birds. On the other hand, startle display function of tufts is also plausible and it is likely that tufts may actually act as a context-dependent multi-function structure, providing camouflage during daytime sleep or rest, but also being actively used to deter predators or mobbers. While our phylogenetically-informed approach lays broad foundations for understanding functionality of owl ear tufts, further experimental studies are required to elucidate tuft role in anti-predatory and anti-mobbing behaviors under different ecological context. We recommend that camouflage function hypothesis should be tested via surveys on owl detectability using vision models consistent with the visual system of potential predators (e.g., [46]), while anti-mobbing function of ear tufts should be assessed via field behavioral experiments with owl models and free ranging passerines (e.g., [10]).

## Supplementary Information

Additional file1 (XLSX 21 KB)

## Data Availability

The datasets used and/or analysed during the current study are available from the corresponding author on reasonable request.
